# Supplementary material for: COZOID: contact zone identifier for visual analysis of protein-protein interactions
Source: BMC Bioinformatics. 2018 Apr 6;19:125. doi: 10.1186/s12859-018-2113-6 (PMC5889581; doi:10.1186/s12859-018-2113-6)
Supplement: Supplementary file 2 — Software build. Executable binary file of the software tool. (ZIP 118,784 kb) [file 12859_2018_2113_MOESM2_ESM.zip › cozoid/java/docs/beansbinding-1.2.1-doc.zip!/org/jdesktop/el/BeanELResolver.BeanProperty.html]

BeanELResolver.BeanProperty (Beans Binding)


---


|  |  |  |
| --- | --- | --- |
| **Overview** \| **Package** \| **Class** \| **Use** \| **Tree** \| **Deprecated** \| **Index** \| **Help** | |  |
| **PREV CLASS**   **NEXT CLASS** | **FRAMES**    **NO FRAMES**     **All Classes** |  |
| SUMMARY: NESTED \| FIELD \| CONSTR \| METHOD | DETAIL: FIELD \| CONSTR \| METHOD |  |


---


## org.jdesktop.el Class BeanELResolver.BeanProperty

```
java.lang.Object
  org.jdesktop.el.BeanELResolver.BeanProperty
```

**Enclosing class:**: BeanELResolver

---

``` protected static final class BeanELResolver.BeanProperty extends java.lang.Object ```

---

**Constructor Summary**

`BeanELResolver.BeanProperty(java.lang.Class<?> baseClass,
java.beans.PropertyDescriptor descriptor)`


| **Method Summary** | |
| --- | --- |
| `java.lang.Class` | `getPropertyType()` |
| `java.lang.reflect.Method` | `getReadMethod()` |
| `java.lang.reflect.Method` | `getWriteMethod()` |
| `boolean` | `isReadOnly()` |

**Methods inherited from class java.lang.Object**

`clone, equals, finalize, getClass, hashCode, notify, notifyAll, toString, wait, wait, wait`

**Constructor Detail**

### BeanELResolver.BeanProperty

```
public BeanELResolver.BeanProperty(java.lang.Class<?> baseClass,
                                   java.beans.PropertyDescriptor descriptor)
```


**Method Detail**

### getPropertyType

```
public java.lang.Class getPropertyType()
```

---


### isReadOnly

```
public boolean isReadOnly()
```

---


### getReadMethod

```
public java.lang.reflect.Method getReadMethod()
```

---


### getWriteMethod

```
public java.lang.reflect.Method getWriteMethod()
```


---


|  |  |  |
| --- | --- | --- |
| **Overview** \| **Package** \| **Class** \| **Use** \| **Tree** \| **Deprecated** \| **Index** \| **Help** | |  |
| **PREV CLASS**   **NEXT CLASS** | **FRAMES**    **NO FRAMES**     **All Classes** |  |
| SUMMARY: NESTED \| FIELD \| CONSTR \| METHOD | DETAIL: FIELD \| CONSTR \| METHOD |  |


---
